# Toward *In Vivo* MRI of the Tissue Proton Exchange Rate in Humans

**DOI:** 10.3390/bios12100815

**Published:** 2022-10-01

**Authors:** Mehran Shaghaghi, Kejia Cai

**Affiliations:** 1 Department of Radiology, University of Illinois at Chicago, Chicago, IL 60612, USA; 2 Department of Biomedical Engineering, University of Illinois at Chicago, Chicago, IL 60607, USA

**Keywords:** proton exchange rate, multiple sclerosis, stroke, pH, oxidative stress

## Abstract

Quantification of proton exchange rate (*k_ex_*) is a challenge in MR studies. Current techniques either have low resolutions or are dependent on the estimation of parameters that are not measurable. The Omega plot method, on the other hand, provides a direct way for determining *k_ex_* independent of the agent concentration. However, it cannot be used for *in vivo* studies without some modification due to the contributions from the water signal. *In vivo* tissue proton exchange rate (*k_ex_*) MRI, based on the direct saturation (DS) removed Omega plot, quantifies the weighted average of *k_ex_* of the endogenous tissue metabolites. This technique has been successfully employed for imaging the variation in the *k_ex_* of *ex vivo* phantoms, as well as *in vivo* human brains in healthy subjects, and stroke or multiple sclerosis (MS) patients. In this paper, we present a brief review of the methods used for *k_ex_* imaging with a focus on the development of *in vivo* *k_ex_* MRI technique based on the DS-removed Omega plot. We then review the recent clinical studies utilizing this technique for better characterizing brain lesions. We also outline technical challenges for the presented technique and discuss its prospects for detecting tissue microenvironmental changes under oxidative stress.

## 1. Introduction

Chemical exchange saturation transfer (CEST) magnetic resonance imaging (MRI) is an emerging imaging technique for noninvasive imaging of the endogenous metabolites in tissue, which includes proteins, peptides, amino acids, and other small molecules. In this MRI technique, the contrast is created by the exchange of the selectively saturated exchanging protons with water protons. The exchanging protons can be those in the tissue metabolites, in their amine –NH_2_, amide –NH, hydroxyl –OH, or sulfhydryl –SH groups. Depending on the abundance of CEST-expressing metabolites in tissue, the exchange rate, and the frequency offset of their exchanging protons, CEST MRI has been tailored for non-invasive imaging of mobile proteins and peptides with amide groups [1,2,3,4,5], liver glycogen [6], cartilage glycosaminoglycans [7], brain glutamate [8,9,10], myo-inositol [11,12], creatine [13,14,15], as well as other metabolites, with a spatial resolution down to sub-millimeter levels. Proton exchange is the fundamental contrast mechanism in CEST MRI. It also plays an integral role in producing other MR imaging contrasts, including T_1_- and T_2_- relaxations, macromolecular magnetization transfer (MT), and exchange-relayed nuclear Overhauser enhancement (NOE) [16].

Despite the importance of proton exchange in various MRI contrasts, *in vivo* quantification and mapping of *k_ex_* remains a challenge and has not been achieved until recently [17]. Current methods for *k_ex_* measurement include MR spectroscopy (MRS), model-based fitting, and exogenous contrast agents. Water exchange (WEX) [18] and the measurement of linewidths [19] are techniques built on MRS which inherently has low resolution and is more suitable for slow exchange rates to avoid signal loss [20]. The method of fitting the Z-spectral to Bloch–McConnell equations for the quantification of *k_ex_* [21] is highly dependent on the initial estimation of parameters that are not measurable. Similarly, novel fingerprinting-based MRI [22,23] has potential for clinical translation but it also relies on simulated datasets from Bloch-McConnell models. Exogenous contrast-based methods [24] require the injection of chemical agents to compete with the background signal. These methods all require knowing the concentration of the agent that is hard to measure. Quantifying exchange using saturation time and saturation power dependencies [20] and their linearized versions [25,26] offer a more straightforward fitting process for *k_ex_* measurement.

The Omega plot method [25], initially developed for paramagnetic CEST (paraCEST) experiments [27], on the other hand, provides a direct way for determining *k_ex_* independent of the CEST agent concentration. In this method, exchange rate values are quantified by fitting the Omega plot, consisting of the inverse of the signal intensity, M_z_/(M_0_−M_z_), as a function of 1/ω^2^ (ω = γB_1_ refers to the saturation pulse strength). However, for *in vivo* tissues, the proton exchanging molecules are endogenous diamagnetic CEST (diaCEST) metabolites [28], with resonance frequencies that are close to water. The small offset frequency of the diaCEST agents, typically within 5 ppm [29], means their signal is affected by the neighboring water direct saturation (DS) signal. It has been demonstrated that by considering the water DS effect and removal of its contribution, the Omega plot method improved the *k_ex_* quantification in solution phantoms [30].

In what follows, we describe the improved Omega plot method, hereafter the DS-removed Omega plot, for *in vivo* tissue *k_ex_* mapping. We then review the studies on the validation of the DS-removed Omega plot method and its applications for healthy human subjects and patients with various pathologies. Studies have demonstrated the diagnostic value of MRI-based *in vivo k_ex_* imaging for assessing lesion activity in neurological diseases such as multiple sclerosis (MS) lesions stratification and monitoring treatment responses in strokes. The findings are suggestive of *k_ex_* MRI as an invaluable imaging biomarker for oxidative stress and related pathologies once it gets confirmed with parallel biopathological studies.

## 2. Methods

### 2.1. Basic Principles

The principle of *k_ex_* MRI using CEST can be explained in a modeled two-pool exchanging system between pool A (such as bulk water protons) and pool B (solute molecules with exchangeable protons) as follows. Low-concentration solute molecules with exchangeable protons (e.g., amide, amine, hydroxyl protons) are selectively irradiated with radiofrequency (RF) irradiation. Continuous RF irradiation reduces the signal of the solute molecules when the rate of saturation is fast compared to the solute’s inherent relaxation rates (R_1_ and R_2_). Due to the proton exchange of the saturation-labeled solute with water protons, the water signal becomes indirectly saturated. Given the low concentration of solute (μM to mM), a single transfer of saturation would be insufficient to show any effect on water protons (~100 M). However, by using a prolonged RF irradiation the saturation of the exchangeable protons in the solute pool can continuously transfer to the water pool by exchanging the saturated protons in the solute pool with the non-saturated protons from the water pool. For the solute protons with a sufficiently fast *k_ex_* and for sufficiently long relaxation times of water, T_1_ and T_2_, continuous RF irradiation (for several seconds) leads to a substantial enhancement of the saturation transfer effect.

The reduction in the signal of the pool B protons due to RF irradiation can be written as

(1) $$m^{B}=\frac{M_{Z,ss}^{B}}{M_{Z,eq}^{B}\;}=1-\alpha$$

where α is the saturation efficiency. For relatively fast exchange rates compared to T_1_ and T_2_ relaxation rates ($k_{ex}\gg R_{1}\;\&\;R_{2}$), the saturation efficiency can be approximated as [20,31,32,33]

(2) $$\alpha =\frac{\omega_{1}^{2}}{\omega_{1}^{2}+k_{ex}^{2}}$$

in which $\omega_{1}=\gamma B_{1}$, where $B_{1}$ is the applied saturation pulse (RF irradiation) power and γ is the nuclear gyromagnetic ratio (1 µT is equivalent to 42.58 Hz for protons). The rate of signal loss in pool A due to saturation transfer from pool B or the rate of the saturation transfer *R_ST_* can be determined by considering these factors: the exchange rate between the pools ($k_{ex}$); the fraction of protons in the solute molecule relative to that of water ($f_{B}$); and the signal of pool B protons ($1-m^{B}$).

(3) $$R_{ST}=k_{ex}\times f_{B}\times \left(1-m^{B}\right)=k_{ex}\times f_{B}\times \alpha =k_{ex}\times f_{B}\times \frac{\omega_{1}^{2}}{\omega_{1}^{2}+k_{ex}^{2}}$$

Concurrently, the reduced signal of pool A protons (due to the exchange mechanism) recovers back because of the inherent spin-lattice relaxation (T_1_-recovery) process with the rate of

(4) $$\left(1-\frac{M_{Z,ss}^{A}}{M_{Z,eq}^{A}}\right)R_{1}^{A}$$

where ($R_{1}^{A}=1/T_{1}^{A}$). Under a steady-state condition, the signal loss balances the signal gain in pool A, and the magnitude of the steady-state signal due to the CEST effect can be described by

(5) $$\frac{M_{Z,ss}^{A}}{M_{Z,eq}^{A}}\times R_{ST}=\frac{M_{Z,ss}^{A}}{M_{Z,eq}^{A}}\times k_{ex}\times f_{B}\times \frac{\omega_{1}^{2}}{\omega_{1}^{2}+k_{ex}^{2}}=\left(1-\frac{M_{Z,ss}^{A}}{M_{Z,eq}^{A}}\right)R_{1}^{A}\;$$

The *k_ex_* can be assessed by noting that this relation for the magnetization signal of the water pool can be rewritten as

(6) $$\frac{M_{Z,ss}^{A}}{M_{Z,eq}^{A}-M_{Z,ss}^{A}}=\frac{R_{1}K_{ex}}{f_{B}}\left(\frac{1}{k_{ex}^{2}}+\frac{1}{\omega_{1}^{2}}\right)$$

This relation (the Omega plot relation) indicates the linear dependency of $y=\frac{M_{z}}{M_{eq}-M_{z}}$ versus $x=\frac{1}{\omega_{1}^{2}}$. Additionally, the x-intercept of the plot of $\frac{M_{z}}{M_{eq}-M_{z}}$ as a function of $\frac{1}{\omega_{1}^{2}}$ (the Omega plot) leads to the *k_ex_* value. Multiplying the Omega plot relation, term by term, by $\omega_{1}^{2}$ gives a similar equation

(7) $$\frac{M_{Z,ss}^{A}}{M_{Z,eq}^{A}-M_{Z,ss}^{A}}\omega_{1}^{2}=\frac{R_{1}k_{ex}}{f_{B}}\left(\frac{\omega_{1}^{2}}{k_{ex}^{2}}+1\right)$$

that can also be used to determine the *k_ex_* value from the x-intercept of the new plot of $y=\frac{M_{z}}{M_{eq}-M_{z}}\omega_{1}^{2}$ versus $x=\omega_{1}^{2}$ due to the linear dependency of y and x. While in either plot the exchange rate can be determined from the x-intercept of the plot, in the second one, the data points contribute to the linear regression with equal weights.

The Omega plot method has been generalized to include multiple proton-exchanging metabolites [17]. It can be shown that in this situation the same relations hold if one considers *k_ex_* as the convoluted exchanging rates of the multiple exchange sources (n), defined as

(8) $$k_{ex}^{2}=\frac{\sum_{1}^{n}\frac{k_{s_{i}}}{f_{i}}}{\sum_{1}^{n}\frac{1}{f_{i}k_{s_{i}}}}=M_{1}\left(\frac{k_{s_{i}}}{f_{i}}\right)\times M^{-1}\left(f_{i}k_{s_{i}}\right)=p^{2}\left(f_{i}k_{s_{i}}\right)=p^{2}\left({\bar{k}}_{ex}\right)$$

in which $k_{s_{i}}$ are the exchange rates of the metabolites with water, $f_{s_{i}}$ are the fraction of their protons relative to that of water, and $M_{1}$ and $M^{-1}$ indicate the arithmetic and the harmonic means, respectively.

In practice, *in vivo* tissue *k_ex_* is a weighted average exchange rate of all exchanging protons contributing to various MRI contrasts, such as semi-solid MT, CEST, and exchange-relayed NOE [16]. The CEST signal due to amide protons (APT) (around 3.5 ppm downfield from the water resonance) is commonly used as the reference signal for *in vivo k_ex_* imaging. Many proteins and peptides contain multiple amide protons at about the same frequency offsets with the cumulative proton concentration of about 70 mM, leading to a large composite resonance [34]. In this way, the detection sensitivity of MRI techniques based on APT CEST corresponds to the millimolar concentration of substances in tissue.

**Figure 1:**
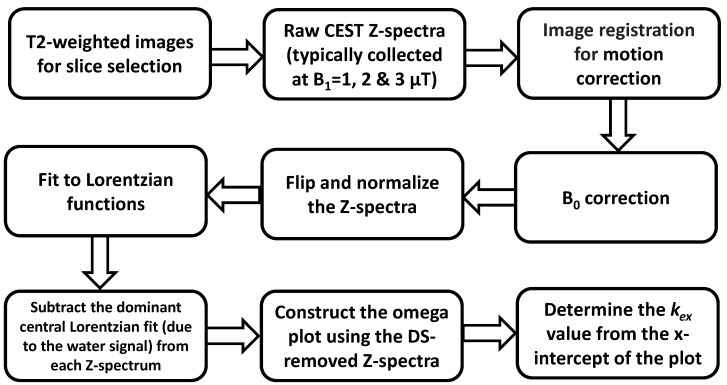
Flowchart of data processing steps in the DS-removed Omega plot method for quantifying *in vivo* tissue *k_ex_*.

In collecting the Z-spectral signal *in vivo*, DS and conventional semi-solid MT occur along with the CEST effect. The water DS effect is particularly dominant when the saturation offset is close to the water proton resonance. The DS effect can thus contaminate the proton exchange rate quantification using the Omega plot, especially for endogenous diaCEST metabolites. These dominant DS effects need to be separated out. This can be conducted using analytical signal removal, which has been attempted via fitting the flipped Z-spectrum to multiple Lorentzian functions. The schematic of the method is outlined as a flowchart in Figure 1, and the details are described in what follows.

### 2.2. DS Removing

To extend the Omega plot analysis for the exchangeable protons whose signal is overlapped by the DS water signal, one may mathematically remove the DS signal from the raw CEST signal by analytical methods. Fitting to Lorentzian functions has been used to analyze the Z-spectral signal as a linear combination of multiple components [35]. This method has already been employed in many studies to fit out background signals from DS and MT and to study the remaining CEST-based signals of interest [13,15,36,37].

Given the prominent DS signal in the Z-spectra, one can readily employ fitting the central portion of the Z-spectrum to Lorentzian functions to compensate for the DS contribution. Each Z-spectrum can be fitted to just two Lorentzian functions: a relatively narrow one, which corresponds to DS, and an overly broad one, corresponding to all the residual signals (containing CEST, NOE, and the semi-solid MT). After removing the fitted Lorentzian function corresponding to the DS signal from the total signal, the residue Z-spectrum can be used for constructing the Omega plots and, subsequently, the exchange rate determination.

In this fitting procedure, the Z-spectra within the ±6 ppm range are normalized to the signal at an offset that represents no saturation (>>10 ppm). Z-spectra are then flipped to be 100 × (1 − M_z_/M_0_) and fitted to a sum of two Lorentzian functions: one (centered around 0 ppm) corresponding to the bulk water signal, and the second (centered about −1.5 ppm) to all the remaining effects (mainly MT from semi-solid components, CEST and NOE, together hereafter referred to as the DS-removed Z-spectrum). In this method, the offset of the fitted water DS peak can be estimated as static field B_0_ map. The *k_ex_* values can then be calculated using the described Omega plot methods based on a set of DS-removed Z-spectra under varied saturation B_1_ power. Exchange rate maps can be constructed following this procedure for each pixel. 

For fitting the Z-spectra to the Lorentzian function and the DS-removal procedure, one can use MATLAB’s nonlinear constrained routine “lsqcurvefit” iteratively with the fitting function

(9) $$Z\left(\omega \right)=100-\overset{2}{\underset{1}{\sum }}L_{n}\left(\omega \right)$$

(10) $$L_{n}\left(\omega \right)=\frac{A_{n}}{1+4{\left(\frac{\omega -\omega_{0,n}}{lw_{n}}\right)}^{2}}$$

where *ω* is the frequency offset relative to water resonance and *A*, *ω*_0_ and *lw* are the amplitude, center frequency offset, and the linewidth of each peak, respectively. Their initial values can be chosen in reference to the values listed in [13]. To allow the fitting parameter to vary from these initial parameters the constraints should be loosely chosen. More details are described elsewhere [17]. It is advisable to start the fitting from the Z-spectrum acquired at the lowest saturation power due to narrow linewidths for each component that are more distinguishable. The fitted parameters can then be used as the starting fitting values for the next Z-spectrum at the higher saturation power.

After the fitting, the DS peak is subtracted from the raw Z-spectra, and the remaining signal at particularly offset (such as +3.5 ppm)

(11) $$Mz\left(\omega \right)=Z\left(\omega \right)-L_{DS}\left(\omega \right)$$

is used for the construction of the Omega plot and determining the *k_ex_* value, as described in the previous section.

Before Z-spectral fitting, one may need to perform motion correction. The motion artifacts due to movements during the data acquisition can be corrected by MATLAB’s intensity-based image registration routine “imregister”. Once a reference image is selected (typically the image collected at a middle offset), the images from each Z-spectrum can be registered to that. Since in each Z-spectrum set the overall intensity of the images changes gradually as the saturation frequency shifts closer to the water resonance, an efficient succession could be that each image gets registered to the image with the adjacent offset, which is assumed to have the most comparable intensity [38].

## 3. Validation

The Omega plot method technique has already been validated with multiple methods, including direct NMR linewidth fitting, as well as fitting CEST spectra to Bloch equations. The results show that it is possible to quantify the *k_ex_* for underlying CEST systems at different temperatures and pH levels [25,30,39]. As mentioned in the introduction, for *in vivo* studies, the overlapping water signal interferes with the signal of the diaCEST metabolites. A DS removal scheme is thus proposed in the DS-corrected Omega plot technique to address this issue. It has been demonstrated that the accuracy of the DS-corrected Omega plot was greatly improved compared to the uncorrected one, using both numerical simulations and experiments on creatine solutions at varying concentrations or pHs [30].

**Figure 2:**
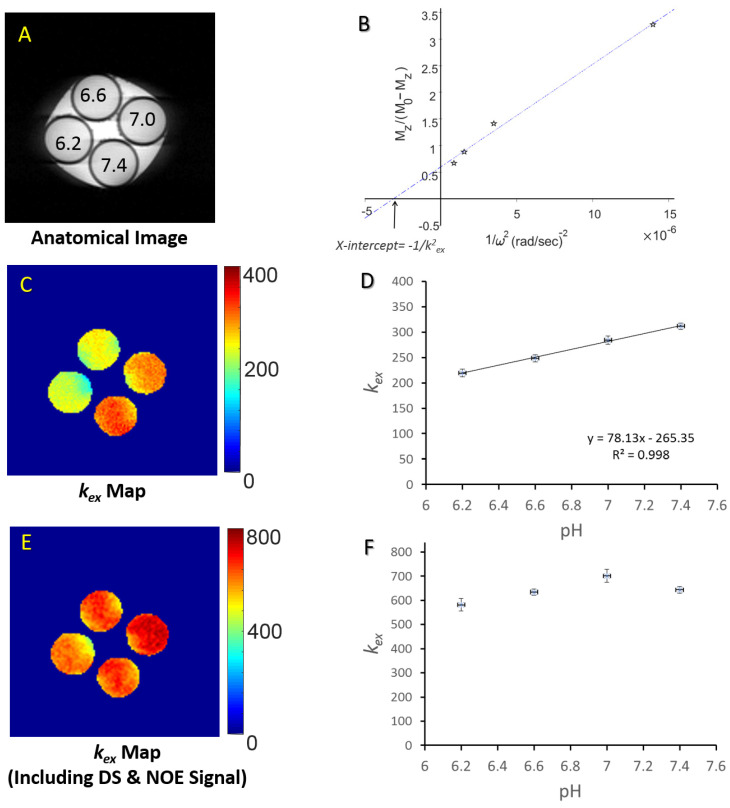
*k_ex_* MRI based on the DS-removed Omega plots implemented on phantom protein solutions containing 20% (*w*/*w*) bovine serum albumin (BSA) in phosphate-buffered saline (PBS) at room temperature with pH titrated to 6.2, 6.6, 7.0 and 7.4. (**A**) T_2_-weighted image of the phantoms (**B**) A typical Omega plot, M_z_/(M_0_ − M_z_) versus 1/ω^2^ (ω = γB_1_), constructed from DS-removed Z-spectra for the pH 6.6 phantom. The linearity of the plot is clear. The x-intercept of the fit to the Omega-plot provides a direct readout of the exchange rate. (**C**) The constructed proton exchange rate maps. (**D**) The proton exchange rate values show that the exchange rate values increase linearly as pH increases. (**E**,**F**) The proton exchange rate values from the raw Z-spectra failed to distinguish different pH and did not show a linear dependency with pH values. (Reprinted with permission from Quant Imaging Med Surg. 2019; 9(10): 1686–96, Figure 5 (CC BY-NC-ND 4.0)).

The applicability of the DS-removed Omega plot for *k_ex_* imaging was also validated using phantoms involving protein solution at varied pH in the physiological range (pH 6.2 to 7.4) as pH is known to change *k_ex_* [17]. The results show that *k_ex_* quantified from the DS-removed plots are linearly correlated with the pH values (R^2^ = 0.998). In comparison, without DS-removing, *k_ex_* showed a reduced linear dependency on pH with a reduced dynamic range due to pH variations (Figure 2). The water DS signal reduces the overall Z-spectral signal and therefore significantly contributes to *k_ex_* evaluation based on Omega plots. Therefore, removing DS increases the sensitivity to detecting *k_ex_* changes, particularly for endogenous metabolites’ exchangeable protons whose resonance offsets are overlapped by the bulk water signal.

## 4. Applications

The added value of *k_ex_* MRI in differentiating the affected brain tissues can potentially serve as a surrogate imaging biomarker for the metabolic changes of the tissue. This can aid in the monitoring and assessment of treatment effects as well as guiding therapies, such as radiotherapy, local chemotherapy, and surgery. The DS-removed Omega plot method has been implemented in several studies for *in vivo* tissue *k_ex_* quantification and mapping of brains of healthy subjects [17], patients with stroke lesions [40], or MS lesions [41].

*k_ex_* MR imaging of the human brain usually included the following acquisitions. T_2_-weighted images were obtained for slice selection. Z-spectral data were then acquired with several saturation powers (B_1_) in the range of 1 to 5 µT (such as B_1_ = 1.5, 2.5 and 3.5 µT) with saturation duration of 1.5 or 2 sec, covering a wide range of saturation offsets from −5 to 5 ppm at an increment of 0.25 ppm or less and a reference image at an offset far from water (such as close to 100 ppm or more).

### 4.1. Mapping the Healthy Human Brain

The applicability of *in vivo k_ex_* mapping studies was first demonstrated in healthy human brains [17]. Ten healthy volunteers (males and females, 22–25 years old) underwent MRI scanning for the described *k_ex_* MRI on a 3.0 T scanner with a 32-channel head coil. The *k_ex_* maps were created using four sets of Z-spectral images acquired with the saturation powers = 1, 2, 3 and 4 µT and saturation duration = 1.5 s on a single slice from the middle brain parallel to the anterior commissure–posterior commissure (AC-PC) line. The quantified *k_ex_* with the DS-removed Omega plot showed significantly higher values in the gray matter (GM) region than in the white matter (WM) region (616 ± 29 vs. 575 ± 20 s^−1^, *p* < 0.001) [17].

The higher exchange rate observed in the GM compared to the WM is most likely the result of differences in their metabolites. This is because the other factors influencing the exchange rate, such as pH and temperature, are not expected to differ significantly. In general, GM has greater metabolic activity than WM. This means it may contain higher levels of metabolites with relatively high exchange rates compared to WM. This study indicates that the *in vivo k_ex_* method can reliably detect variations in the metabolite profile when there is no significant variation in pH and temperature.

One should note that the calculated exchange rate in tissue is in general a weighted average of all saturation transfer exchanging proton species that contribute to the Z-spectral signal. *In vivo*, there are many endogenous metabolites, which undergo many saturation transfers and proton exchanges at different offset frequencies and different exchange rates with broad and overlapping peaks. The downfield side of the Z-spectrum has contributions from various saturation transfer sources, including semi-solid macromolecules (the MT effect) and various exchangeable labile proton species present *in vivo*. Furthermore, metabolites have different *k_ex_* and each gets optimally saturated under a different saturation power. These considerations and the fact that Omega plots require a set of saturation powers indicate that the quantified *k_ex_* in tissue is a weighted average of all contributing metabolites’ *k_ex_* from all proton-exchanging mechanisms.

### 4.2. Detecting the Metabolic Disturbance of Ischemic Stroke Tissues

**Figure 3:**
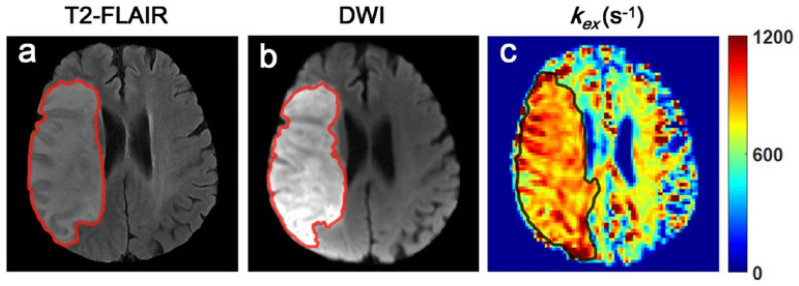
Different MRI images from a representative ischemic stroke patient showing the infarct lesion area. (**a**) T_2_-weighted fluid-attenuated inversion recovery (T_2_-FLAIR) image. (**b**) DWI. (**c**) *k_ex_* MRI. The *k_ex_* map reflects a larger extent of the infarct lesion with an elevated signal. (Reprinted by permission from J Neurosci Methods. 2020; (346), 108926. Figure 4).

*In vivo k_ex_* MRI based on the improved Omega plot has been implemented to map the brains of ischemic stroke patients [40]. In a study of 23 patients with ischemic stroke, *k_ex_* MRI maps were constructed using three sets of Z-spectra (B_1_ = 1.5, 2.5 and 3.5 µT) and saturation duration = 1.5 s, along with the other conventional contrast, including diffusion-weighted imaging (DWI), CEST, and semi-solid MT MRI. The derived *k_ex_* maps differentiated infarcts from the contralateral normal brain tissues with significantly increased *k_ex_* (893 ± 52 s^−1^ vs. 739 ± 34 s^−1^_,_ *p* < 0.001). The *k_ex_* maps were also found to differ from the conventional contrast MRIs. In the *k_ex_* maps, the infarct lesions displayed considerably larger areas than that delineated in DWI (3.69–297%, mean and standard deviation 43.6 ± 67.7%, *p* < 0.05) for all cases (Figure 3). In addition, the *k_ex_* maps also showed different lesion contrast compared to CEST and MT.

In stroke clinics, the definition of ischemic penumbra is useful in the decision for recanalization therapy. The observed mismatch between *k_ex_* and DWI maps suggests that *k_ex_* MRI may serve as a novel and independent MRI contrast for assessing affected brain tissues in stroke patients and defining ischemic penumbra. Ongoing investigations with longitudinal and histological studies will validate this concept.

### 4.3. Detecting and Grading MS Lesions

The described method of *in vivo k_ex_* MRI has been performed for MS patients and its potential value for staging clinical MS lesions has been evaluated [41]. MS is an autoimmune neurodegenerative disease that is a leading cause of neurologic disability in young adults. It involves the chronic inflammation of the central nervous system, characterized by lesions in multiple tissues, especially in demyelination of the white matter that may later progress to axonal damage. The diagnosis of MS is currently based on the McDonald criteria [42], in which MRI is an essential part.

MS lesions can be classified into five pathological stages: early active, late active, smoldering, inactive, and shadow plaques [43]. The ability to risk-stratify MS lesion activity would help predict prognosis and guide treatment. As part of this effort, PET tracers are being developed and tested for imaging demyelination and inflammation in MS [44], including MeDAS [45] for myelination, Pittsburgh compound B (PiB) [46] for amyloid, translocator protein 18 (TSPO) [47] for neuroinflammation. However, each tracer has different binding specificity and half-life and the use of PET in MS even in the research setting is still in its infancy [44]. MRI methods do not use ionizing radiation and can be easily incorporated into clinical protocols. With considerable sensitivity [48,49], conventional T_1w_&T_2w_ MRI methods detect MS lesions as hypointense black holes (axonal loss) [50] and with hyperintensity (edema or demyelination) [51], respectively. Through the measurement of blood–brain barrier breakdown, Gd-enhanced MRI is commonly employed to discern different stages of the lesions in an MS patient. Gadolinium (Gd) enhancement on postcontrast T_1_-weighted images (T_1_WI), signals blood–brain barrier disruption and active inflammation [43].

Gd enhancement in MS lesions, however, has a narrow time window, so underestimating the lesion’s activity is common. Individual new lesions show the average duration of Gd enhancement in about 3 weeks [52], and the vast majority of Gd enhancements disappear within 6 months [53]. Later, when MS lesions turn into non-enhanced lesions, their stages cannot be identified. It has been known that after Gd-enhancement resolves, inflammation persists in some lesions [54,55,56]. These non-enhancing lesions can be categorized according to the presence or absence of ongoing inflammation into chronic active lesions (slowly expanding lesions) and chronic inactive lesions [56,57,58]. As a result, the activity of the lesions is often underestimated with only Gd-enhanced MRI evaluations. This makes developing further MRI biomarkers to improve the characterization of MS lesions highly desirable. There are several emerging MRI methods being investigated for MS studies, including diffusion tensor or kurtosis imaging for changes in water diffusion in myelin [59,60,61], multicomponent T_2_ mapping [62,63,64], and ultrashort or zero echo time (UTE) MRI [65,66] for myelin water, and quantitative susceptibility mapping (QSM) [67] sensitive to iron deposition.

Sensitive to the myelin lipid content, MT MRI [68,69] has been used as an MR method to detect demyelination in MS [70,71]. Additionally, metabolic changes used as early biomarkers of MS have been evaluated with proton MRS [72,73,74], however, with limited sensitivity [75]. Molecular CEST MRI, on the other hand, produces high-resolution metabolite maps with hundreds to thousands of times amplified sensitivity compared to MRS [75]. This includes the described DS-removed Omega plot method for *k_ex_* mapping.

**Figure 4:**
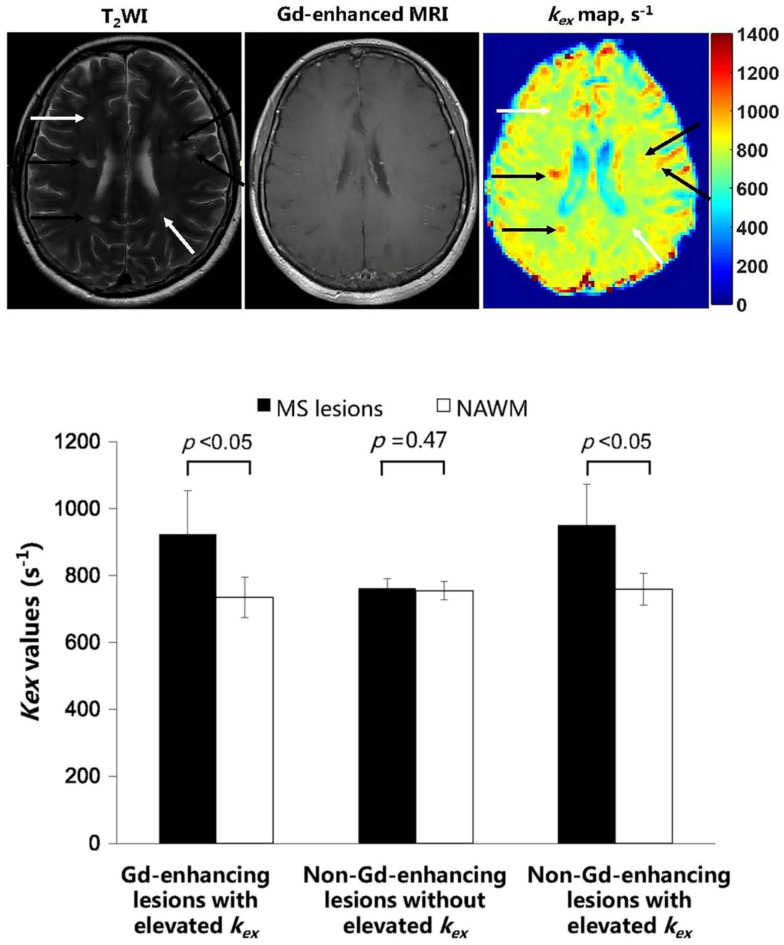
A total of 153 MS lesions in 16 MS patients were identified on *k_ex_* images and compared with those on T_2_ weighted and gadolinium (Gd) enhanced MR images. Top row: A representative case, in which among the 6 lesions identified on the T_2_WI slice none showed Gd enhancement while 4 of them (black arrows) showed elevated *k_ex_*. Bottom row: Categorization of all studied 153 MS lesions based on Gd-enhancement and *k_ex_* MRI put them in three distinguished patterns. While all the Gd-enhancing lesions showed significant elevation in *k_ex_* value compared to the normal-appearing white matter (NAWM), the non-Gd-enhancing lesions showed either a significant *k_ex_* elevation (76%) or no *k_ex_* elevation (24%) compared to NAWM. (Reprinted from J Magn Reson Imaging. 2020; 53(2), 408–415, Figures 4 and 5).

*In vivo k_ex_* MRI was implemented in MS in this regard. Brain *k_ex_* MRI in MS patients was performed with the described DS-removed Omega plot method and showed a high correlation to Gd-enhanced images, with increased *k_ex_* signal in all the Gd-enhanced lesions [41]. In this study, 16 MS-diagnosed subjects (7 male, 9 female) underwent a brain MRI on a 3 T clinical scanner. *In vivo k_ex_* maps were generated by the DS-removed Omega plot constructed from five different saturation powers (B_1_ = 1, 2, 3, 4 and 5 µT) and saturation duration = 1.5 s. Of all 153 MS lesions, 78 (51%) lesions were Gd-enhancing and 75 (49%) were Gd-negative. All 78 Gd-enhancing lesions showed significantly elevated *k_ex_* values compared to normal-appearing white matter (NAWM) (924  ±  130 s^–1^ vs. 735  ±  61 s^–1^, *p*  <  0.05). For the 75 Gd-negative lesions, 18 lesions (24%) showed no *k_ex_* elevation (762  ±  29 s^–1^ vs. 755  ±  28 s^–1^, *p* = 0.47) and 57 (76%) showed significant *k_ex_* elevation (950  ±  124 s^–1^ vs. 759  ±  48 s^–1^, *p*  <  0.05) compared to NAWM (Figure 4).

These initial findings on *k_ex_* MRI of MS lesions suggest the unique potential of *k_ex_* MRI as a valuable imaging biomarker for characterizing MS lesion activity of Gd-negative lesions. Generally, Gd-enhanced MRI shows limited correlation with clinical disability [76]. It has been shown that some recently developed and most chronic MS lesions are Gd-negative [52,53]. In addition, gadolinium administration has complications [77] with potential brain accumulation [78]. The results suggest that *k_ex_* mapping may be useful in separating slowly expanding MS lesions (chronic active lesions) from inactive ones. Hence, as an endogenous MRI contrast, *k_ex_* MRI could be an alternative technique to the positron emission tomography (PET) with 11C-(R)-PK11195 (targeting activated microglia or macrophages) [44] or susceptibility weighted imaging (SWI, targeting abnormal iron deposition) [79] that have been proposed for differentiation of non-enhancing MS lesions.

## 5. Discussion

We reviewed the studies demonstrating the feasibility of mapping the proton exchange rate *in vivo* with Omega plots using DS-removed Z-spectral signals. RF irradiation is used to implement saturation in this CEST MR imaging technique, which is then transferred to the water protons via the ubiquitous proton exchange. The drop in the water signal due to this transfer of the saturation is detected and the physical parameters get inferred from the transferred saturation. Indeed, the name of the process, i.e., chemical exchange saturation transfer (CEST), clearly described the basics of the technique, which is based on the saturation that is transferred via the chemical exchange process. The basic principle of the CEST technique has been described in several excellent reviews published in recent years [29,33,80,81]. In short, the contrast mechanism in CEST imaging involves the irradiation of RF radiation to produce saturation. As a result of the saturation transfer, MR imaging contrast is produced which enables quantification of exchange-related parameters. Proton exchange is a physical phenomenon that is constantly happening between exchangeable protons in the metabolites and the water protons regardless of whether the saturation RF is “no” or “off”.

In *k_ex_* MRI, the contrast is generated primarily from the *k_ex_* of the endogenous metabolites in the tissue. As a physical parameter, *in vivo k_ex_* may reflect variation in the pH, temperature, reactive oxygen species (ROS) activity, or the profile of tissue metabolites containing slow or fast exchangeable protons [82]. To explain the *k_ex_* variations in the *in vivo* studies, one needs to discern the factors which can explain the observed *k_ex_* variation.

In the ischemic stroke tissues, *k_ex_* MRI mapping presented a larger area of the infarct lesions in the *k_ex_* maps compared to in the DWI maps [40]. This disparity reflects the distinctive contrast mechanism of *k_ex_* imaging as compared to conventional MRI. The observed increased *k_ex_* in the infarct lesions due to ischemic stroke cannot be attributed to factors such as increased pH, temperature, or metabolite changes. It has been shown that following an ischemic stroke, enhanced anaerobic metabolism leads to tissue acidosis and hence a pH reduction in the damaged tissues [83], which would be reflected as a decrease in the *k_ex_* value. Furthermore, the brain temperature is normally well controlled and its variation in the ischemic brain is reported to be within 1 °C [84]. Additionally, it has been shown that during the initial stage of the stroke (<24 h), some minor amino acids with generally higher exchanging protons show an elevation in the tissue [85,86,87]. This, however, should have already subsided since the majority of small metabolites with generally higher exchanging protons are reported to decrease after 24 h [88,89]. This reduction in small metabolites with a relatively faster proton exchange rate compared to macromolecules should result in a reduction in the tissue *k_ex_*.

The elevated *k_ex_* observed in ischemic tissues should be attributed to other factors. Recent studies have shown that the production of ROS can increase the *k_ex_* of tissue metabolites [82,90,91]. During cerebral ischemia, an imbalance occurs between the production of ROS and the antioxidant capacity. This results in excessive ROS production that directly or indirectly contributes to oxidative damage and eventually cellular damage and death [92]. Thus, the pronounced increase in *k_ex_* in the infarct region could be due to elevated ROS production. The hypothesis, however, needs to be further investigated via invasive measurements of tissue ROS in experimental animal models.

Similarly, the observed increases in *k_ex_* values in the MS lesions [41] are suggested to be the result of ROS overproduction and elevated oxidative stress associated with MS pathogenesis [93]. With the potential for targeting ROS [82,90], *k_ex_* MRI may further characterize Gd-negative lesions based on lesion activity. Additional validation is necessary with pathological studies on preclinical MS models.

## 6. Challenges and Opportunities

There are several challenges associated with the described Omega plot-based *k_ex_* imaging, some of which are similar to those associated with other conventional Z-spectrum-based imaging. These techniques in general require a long saturation time to fulfill the steady-state assumption [25], which in theory calls for RF irradiation for as long as ≥ 10 s, potentially leading to a high specific absorption rate (SAR) exposure. To avoid the potential risk of high SAR or RF heat deposition due to the prolonged scanning times, in the clinical settings, the steady-state saturation required by the Omega plot was approximated with a 1–2 seconds-long saturation pulse or pulse train. According to simulations with Bloch-McConnell equations, applying 1.5 s of saturation can result in ~65% of the steady-state saturation when B_1_ = 1 µT and 99% of the steady-state value when B_1_ = 4 µT, for the *k_ex_* values in the 30–3000 s^−1^ range [17].

Another issue is the length of the scanning. The described Omega plot-based *k_ex_* imaging requires multiple Z-spectral acquisitions, which increases the total scanning time. Given the linearity of the Omega plot, the scanning time may be reduced by acquiring two Z-spectra. This has been shown in the healthy brain study that the *k_ex_* maps constructed with only two Z-spectra showed very similar values to those calculated using 4 Z-spectra [17]. The scanning time may be further reduced by decreasing the number of offsets in the Z-spectrum and by implementing fast-reading pulse sequences.

In the described *k_ex_* imaging based on the DS-removed Omega plot, we do not expect artifacts due to the inhomogeneity of B_0_ since the fitting process automatically corrects B_0_ variations. Having a homogeneous B_1_ field over the field of view (FOV), however, is an instrumental factor in producing an accurate *k_ex_* map. The determination of *k_ex_* is based on the X-intercept of the Omega plot, which depends on the B_1_ values of the RF saturation pulse. Inhomogeneity of B_1_ field strength can directly affect the Omega plot intercept and accordingly the *k_ex_*. Therefore, aiming for a homogenous applied RF pulse over the field of view has a significant role in an accurate evaluation of the *k_ex_* variation. Otherwise, B_1_ correction is necessary by acquiring a B_1_ map [8] and using corrected B_1_ values for constructing Omega plots.

Another potentially challenging issue for the *k_ex_* MRI based on the DS-removed Omega plot is the quantification of the DS signal. The Lorentzian fitting of the DS spectrum may not always be sufficiently accurate particularly when saturation power is high and under low field strength. It is still needed to study the interaction of DS with the proton-exchanging mechanisms that are close to water resonance to improve the DS quantification.

In the above-reviewed *in vivo k_ex_* MRI studies, the implementation of the DS-removed Omega plot was done at clinical 3 T scanners. The DS-removed Z-spectra contain a large contribution from MT due to semi-solid macromolecules, which can contribute to the calculated *k_ex_*. Furthermore, at 3 T field strength, the individual CEST-expressing metabolites have broad and overlapping peaks in the Z-spectrum. Therefore, the calculated *k_ex_* in tissue is a weighted average from the various saturation transfer sources, including semi-solid MT associated with macromolecules and CEST effects from various mobile proteins, peptides, and small metabolites. The described method for determining *k_ex_*, however, can be improved by using the Z-spectra collected at higher field strengths. With the better resolved Z-spectra at higher fields (such as at 7 T or 9.4 T), Z-spectral fitting can be utilized to separately quantify individual proton-exchanging mechanisms [13,37,94,95], such as semi-solid MT, creatine CEST at 2 ppm, APT, and relayed NOE, etc. Using the signal of each exchangeable proton group, the Omega plot can be constructed accordingly for determining *k_ex_* more specifically for each proton-exchanging component.

## 7. Conclusions

The Omega plot method is known as a direct MR method for determining *k_ex_* independent of the CEST agent concentration. However, the use of this method is limited to in vitro studies and paramagnetic CEST experiments. This method can be improved by removing the obtrusive water DS contribution from the MR signal and can be applied for *in vivo* studies involving diaCEST metabolites. *k_ex_* MRI based on the DS-removed Omega plot is an emerging technique for mapping the proton exchange rate of tissue metabolites *in vivo*. Proton exchange is an invaluable biophysical parameter that can reflect variations in the imaging milieu due to factors such as metabolite changes, pH, temperature, and ROS overproduction. In this MRI technique, the contrast is generated by endogenous metabolite species with exchangeable protons. Therefore, it does not require exogenous contrast agent injection.

The technique has shown promise for several practical applications, including but not limited to, imaging of ischemic stroke and MS brain. In studies of brain lesions due to ischemic stroke, *k_ex_* MRI presented an improved definition of ischemic penumbra. The published data from clinical MS patients demonstrated that *k_ex_* MRI further differentiates the Gd-negative lesions into *k_ex_* positive and negative groups. These findings suggest the diagnostic value of *k_ex_* MRI for assessing the lesion activity and its potential in enhancing the sensitivity and specificity for lesion stratification, as well as monitoring treatment responses. It was suggested that increased proton exchange promoted by ROS overproduction could be a major contribution to the tissue *k_ex_* elevation. *k_ex_* MRI therefore might be a valuable imaging biomarker for oxidative stress and related pathologies. Once validated with further biopathological studies, *in vivo k_ex_* imaging will have broad clinical applications for the diagnosis and treatment of neurological diseases.
